# Unveiling the landscape of prokaryotic global regulators through deep protein language models

**DOI:** 10.1128/msystems.00950-25

**Published:** 2025-11-24

**Authors:** Jianing Geng, Jiang Wu, Sainan Luo, Dongmei Liu, Jingyi Nie, Guomei Fan, Qinglan Sun, Songnian Hu, Linhuan Wu

**Affiliations:** 1 State Key Laboratory of Microbial Diversity and Innovative Utilization, Institute of Microbiology, Chinese Academy of Sciences 85387, Beijing, China; 2 China National Microbiology Data Center (NMDC), Chinese Academy of Sciences, Beijing, China; 3 University of Chinese Academy of Sciences, Beijing, China; Wageningen University, Wageningen, the Netherlands

**Keywords:** global regulators, transcription factors, prokaryotic genomes, deep learning, protein language models, synthetic biology, regulatory networks

## Abstract

Global regulators (GRs) are key transcription factors that orchestrate the expression of multiple genes, playing essential roles in stress responses, virulence, secondary metabolism, and antibiotic resistance—traits that make them powerful tools for synthetic biology applications. However, conventional approaches often fail to detect remote homologs and novel GR types, limiting our understanding of their regulatory diversity and evolutionary dynamics across prokaryotes. Here, we present a large-scale, protein language model-driven framework to systematically chart the global regulatory landscape across 14,800 bacterial and archaeal type strain genomes—the most taxonomically diverse prokaryotic data set analyzed to date. Using a deep learning-based GR identification model trained on 74,872 curated GR sequences, we systematically identified over 270,000 GR-like proteins, including 173,256 homologs of 214 experimentally validated GR types, 52 putative GR types, and 76,113 proteins of unknown function. This model demonstrated high sensitivity and generalization capability, enabling the discovery of remote homologs and cryptic regulators beyond the reach of similarity- or domain-based methods. This expanded GR catalog revealed lineage-specific distribution patterns, functionally diverse regulons with both conserved and niche-specific targets, and hierarchical cross-regulatory networks with shared and phylum-enriched hubs. By unveiling the hidden diversity and evolutionary structure of prokaryotic global regulators, this landscape of GRs provides foundational insights into microbial gene regulation and offers a powerful toolkit for the rational design of tunable, modular, and orthogonal genetic circuits in synthetic biology.

**IMPORTANCE:**

GRs are master transcriptional regulators critical for microbial adaptation, stress tolerance, and metabolic control, and they serve as valuable components for synthetic biology. However, a comprehensive understanding of GR diversity and function across the prokaryotic domain has remained elusive due to the limitations of traditional detection methods. In this study, we developed a deep learning-based identification framework and applied it to 14,800 bacterial and archaeal type strain genomes, resulting in the discovery of over 270,000 GR-like proteins, including dozens of novel types. This work provides a comprehensive landscape of prokaryotic global regulators, revealing lineage-specific distribution patterns, both conserved and specialized regulons, and modular cross-regulatory network architectures. These insights not only deepen our understanding of transcriptional regulation in microbial evolution and ecology but also provide a scalable resource for the rational design of regulatory systems in synthetic biology.

## INTRODUCTION

Global regulators (GRs) are essential transcriptional controllers that coordinate the expression of multiple genes or entire regulatory networks in response to environmental and physiological signals (1–3). They allow prokaryotes to rapidly adapt to fluctuations in nutrient levels, oxygen availability, pH, and temperature (4). For instance, the cAMP receptor protein (CRP) modulates alternative carbon source utilization under glucose-limiting conditions, enhancing survival (5, 6). GRs also activate protective responses against oxidative and osmotic stress (7, 8), and in pathogenic bacteria, they regulate critical functions including virulence, secondary metabolite production, and antibiotic resistance. Notable examples include *AfsR* in *Streptomyces* (activates antibiotic biosynthesis) (9), *MarA* in *Escherichia coli* (controls multidrug resistance genes) (10, 11), and the *PhoP*/*PhoQ* system in *Salmonella* (regulates virulence under magnesium limitation) (12). In synthetic biology, GRs are increasingly used to build adaptive circuits that precisely control metabolic pathways and enhance target compound production (13–15). Engineering GRs has also enabled the activation of silent biosynthetic gene clusters—so-called “microbial dark matter”—as demonstrated with *AdpA* and *CRP* in *Streptomyces* (16–18). These applications highlight GRs as critical components of microbial physiology, ecological adaptation, and biotechnology.

Conventional GR identification methods—such as electrophoretic mobility shift assays, DNase I footprinting (19), and chromatin immunoprecipitation sequencing (ChIP-seq) (20, 21)—are labor-intensive and typically focus on individual organisms, offering only piecemeal insights into GR diversity (22, 23). Innovations like ChIP-exo and ChIP-mini have improved resolution and enabled GR profiling in low-biomass samples (e.g., intracellular pathogens) (24, 25), but they remain impractical for large-scale or cross-species analyses.

Computational approaches, including sequence similarity and domain-based annotations, typically rely on predefined identity thresholds (26–28). However, GRs often retain structural homology despite low sequence similarity (29), making such methods prone to false negatives. To address this, machine learning (ML) has been increasingly applied to identify regulatory proteins. Traditional ML classifiers such as support vector machines, random forests (RFs), and gradient boosting have been successfully used in related tasks such as genome mining and biosynthetic gene cluster prediction (30). More recently, deep learning frameworks, including convolutional and recurrent neural networks, have been developed for predicting bacterial directly from sequence data, as exemplified by PredicTF (31). In parallel, advances in (pLMs) have enabled the extraction of structural and functional features from raw sequences, supporting high-performance predictions of protein structure (32, 33), remote homology (34), and functional classification (35), and can even support synthetic protein design (36). Together, these diverse ML strategies may provide complementary avenues for mining novel GRs and reconstructing their regulatory networks.

Building on these capabilities, we developed a machine learning pipeline integrating pLM-derived features to identify GRs in 14,800 type strain prokaryotic genomes (37). This approach identified 173,256 additional putative GR homologs corresponding to the 214 experimentally validated GR types. In addition, it uncovered 100,694 GR-like proteins, including 52 putative GR types, 76,113 proteins of unknown function, and 18,815 proteins assigned to known GR families. These findings have been integrated into the Prokaryotic Global Regulatory Resource (PGRR)—a publicly available database that provides comprehensive analysis of GR sequences, functional annotations, and regulatory networks. PGRR serves as a valuable platform for advancing our understanding of prokaryotic transcriptional regulation and supports the rational design of regulatory circuits for synthetic biology and microbial engineering.

## RESULTS

### Identification of homologous proteins for experimentally validated GRs

**Fig 1:**
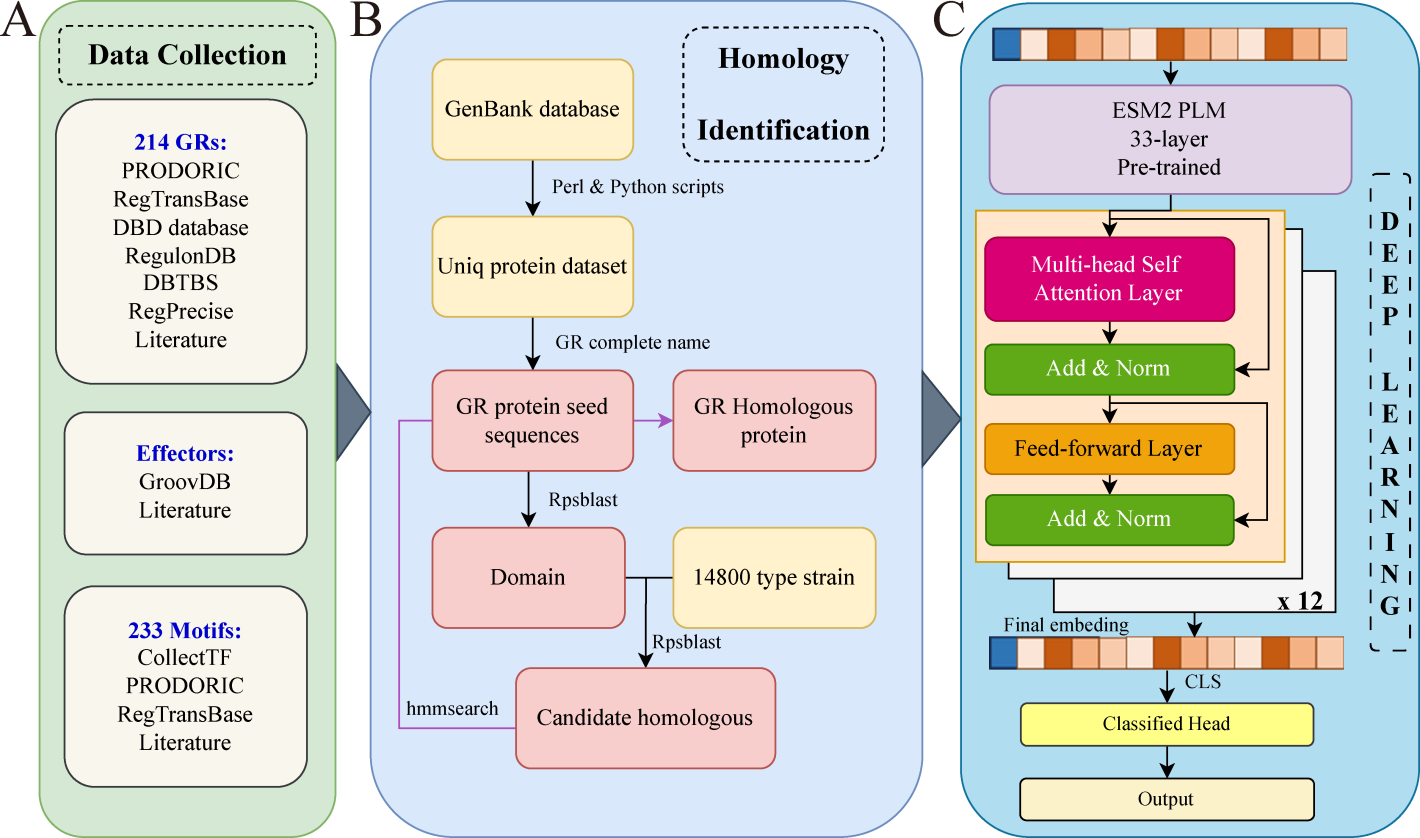
Workflow for the identification and characterization of global regulators (GRs). The pipeline integrates three key components: (**A**) literature and database curation to compile a reference GR data set, (**B**) homology-based identification using Reverse Position-Specific BLAST (RPS-BLAST) and HMMER to detect GR homologs, and (**C**) deep learning-based classification using the ESM protein language model, a GR-specific transformer encoder, and a final prediction head.

To systematically expand the repertoire of GRs, we developed an integrated strategy that combines curated knowledge with large-scale comparative genomics (Fig. 1A and B). Unlike conventional single-database or sequence-similarity approaches, our framework unifies multiple curated GR resources with literature-mined regulators and leverages both domain-based and hidden Markov model (HMM)-based searches across 14,800 type strain genomes. This design enabled the recovery of 107,335 homologous proteins corresponding to 214 experimentally validated GRs, spanning 11,971 species, 2,815 genera, and 47 phyla. By anchoring each GR family to experimentally supported seed sequences while scaling to a phylogenetically broad genome set, the strategy not only maximizes sensitivity for remote homolog detection but also ensures systematic coverage across the prokaryotic tree of life. These homologous proteins further serve as reliable seed sequences for downstream artificial intelligence (AI)-based prediction, facilitating both the discovery of additional GR homologs and the identification of putative novel GR families.

### Prediction of GR proteins based on machine learning

#### Model construction and evaluation

**Fig 2:**
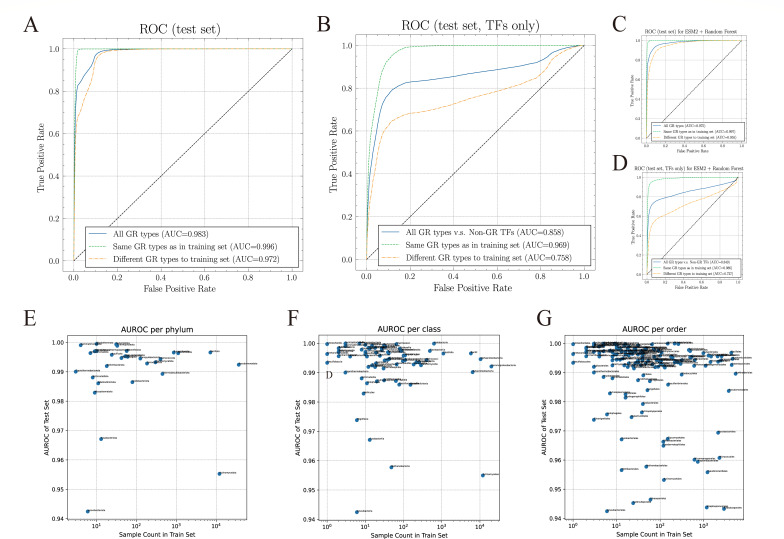
Model performance in classifying global regulators. (**A**) Receiver operating characteristic (ROC) curves for the full test set, showing model performance across all GR types (blue), same GR types as in training set (green), and different GR types to training set (red). (**B**) ROC curves for a subset of the test set using only transcription factors (TFs) as negative examples. The model distinguishes GRs from closely related TFs with high accuracy for known GR types but shows reduced performance for novel types. (**C**) Comparison of ROC curves between the GR-specific transformer model (blue) and the baseline vanilla ESM2 + random forest model (orange) on the full test set. (**D**) Comparison of ROC curves between the GR-specific transformer model (blue) and the baseline vanilla ESM2 + random forest model (orange) using TFs as negative examples. (**E**) Area under the receiver operating characteristic curve (AUC) values calculated at the phylum level. Each dot represents a phylum, with no correlation observed between training data abundance and model performance. (**F**) AUC values calculated at the class level, showing consistently high performance across well-represented and underrepresented taxa. (**G**) AUC values calculated at the order level, further confirming that model performance is not biased by uneven training data distribution.

Based on the GR homologs identified through the bioinformatics pipeline described above, we developed a deep learning classifier integrating ESM2 embeddings with a GR-specific transformer encoder (Fig. 1C). The training data set included 74,872 protein sequences from 142 types of GRs. The negative data set comprised 2,151,890 sequences representing 16 types of functional proteins, including 58,624 sequences annotated as transcription factors. The model was trained using an 8:1:1 split for training, validation, and test data sets, respectively. Model performance was evaluated on a test set consisting of 24,207 GR sequences spanning 169 types of GRs. Of these, 16,732 protein sequences from 27 types of GRs were excluded from the training phase to assess the model’s generalization ability, while the remaining 7,475 protein sequences were from the test data sets of the original 142 types of GRs. The final model demonstrated strong classification performance across all 169 types of GRs (area under the receiver operating characteristic curve [AUC] = 0.983, accuracy = 0.905) and maintained high predictive power on unseen types of GRs (AUC = 0.972; Fig. 2A; Table 1). In a more challenging task distinguishing GRs from other transcription factors, the model sustained high performance for known GRs (AUC = 0.969) but showed reduced predictive accuracy for novel types of GRs (AUC = 0.758, Fig. 2B).

**TABLE 1:** Performance metrics of the GR prediction model across different test subsets^*a*^

Parameter	All–all	All–TF	142GR–all	142GR–TF	27GR–all	27GR–TF
Precision	0.967	0.736	0.968	0.766	0.956	0.697
Sensitivity	0.841	0.841	0.998	0.998	0.701	0.701
Specificity	0.971	0.699	0.967	0.695	0.967	0.696
Accuracy	0.905	0.770	0.982	0.846	0.833	0.698
F1	0.899	0.785	0.983	0.867	0.809	0.699

^*a*^ “All GRs” refers to the full set of 169 GR types in the test data set. “142 GR types” are GRs included in the training set; “27 novel GR types” are held-out types not seen during training. “TFs” refers to non-GR transcription factors used as negative controls. The model demonstrates high precision and generalizability across both seen and unseen GR types, with slightly reduced performance on GR–TF comparisons.

To further benchmark our model, we trained a baseline classifier combining vanilla ESM2 embeddings with an RF. Although this model achieved reasonable performance (overall AUC = 0.975), it showed a stronger trend toward overfitting existing GR types and a larger performance drop on unseen GR types (AUC = 0.955 vs 0.972 for our model, Fig. 2C and D). These results confirm that integrating ESM2 embeddings with a GR-specific transformer encoder improves generalization and robustness compared to simpler classifiers.

To assess whether the uneven distribution of GRs across taxa could be influenced by biases in the training data set, we further examined the relationship between the number of training sequences and model performance. Specifically, we calculated the AUC for predictions at the phylum, class, and order levels and compared these values against the abundance of training data within each group. As shown in Fig. 2E through G, the AUC values remained consistently high across both well-represented and underrepresented taxa, and no significant correlation was observed between training data size and predictive accuracy. These results indicate that the performance of our model is not dependent on the richness of order *Enterobacterales* or other dominant groups in the training data but rather reflects a robust generalization across diverse phylogenetic lineages.

#### AI mining of GRs

Using the trained model, we conducted a large-scale prediction of GRs across 14,800 bacterial and archaeal type strain genomes. This analysis identified 173,256 additional homologous proteins associated with the original 214 types of GRs, along with 100,694 newly predicted GR-like proteins. Among the AI-mining GR sequences, 18,815 sequences were homologs assigned to 88 known GR families; 5,766 sequences were classified into 52 putative GRs; and 76,113 were annotated as proteins of unknown function greatly expanding the known diversity of GRs (Table 2). Notably, the 76,113 proteins of unknown function represent a substantial pool of potentially novel GRs that merit further functional investigation.

**TABLE 2:** Summary of homolog and AI-mining GR statistics across prokaryotic taxa^*d*^

Method	Type	Protein	Species	Genus	Family	Order	Class	Phylum	Domain
Bioinformatics	214	107,335	11,971	2,815	606	247	114	47	2
AI mining	100^*a*^	173,256	3,695 (944)	1,065 (87)	247 (6)	91 (0)	32 (0)	15 (0)	2 (0)
	88^*b*^	18,815	3,300 (891)	978 (80)	232 (5)	87 (0)	31 (0)	15 (0)	2 (0)
	52^*c*^	5,766	2,010 (484)	264 (32)	109 (1)	51 (0)	18 (0)	9 (0)	2 (0)
	Unknown function	76113	3,629 (925)	1,050 (85)	245 (6)	91 (0)	32 (0)	16 (0)	2 (0)

^*a*^ GR types overlapping with 100 of the original 214 experimentally validated GRs.

^*b*^ Predicted proteins assigned to 88 known GR families.

^*c*^ Predicted proteins representing 52 putative GRs.

^*d*^ Values in parentheses indicate the number of newly identified taxa not previously associated with GRs in the curated data set.

The 18,815 sequences assigned to known GR families included prominent groups such as Fur (2,353 sequences), DtxR (2,351 sequences), and LysR. In addition, 5,766 sequences were classified as 52 AI-mined GR groups with annotation information (Table S2). Specifically, 26 groups have been explicitly reported as GRs in the literature, 13 groups were inferred as GRs based on AI prediction and target gene prediction (≥10 predicted target genes across at least two genomes), and the remaining 13 groups were identified solely by our AI-based model prediction.

### Uneven distribution and phylogenetic patterns of GRs

**Fig 3:**
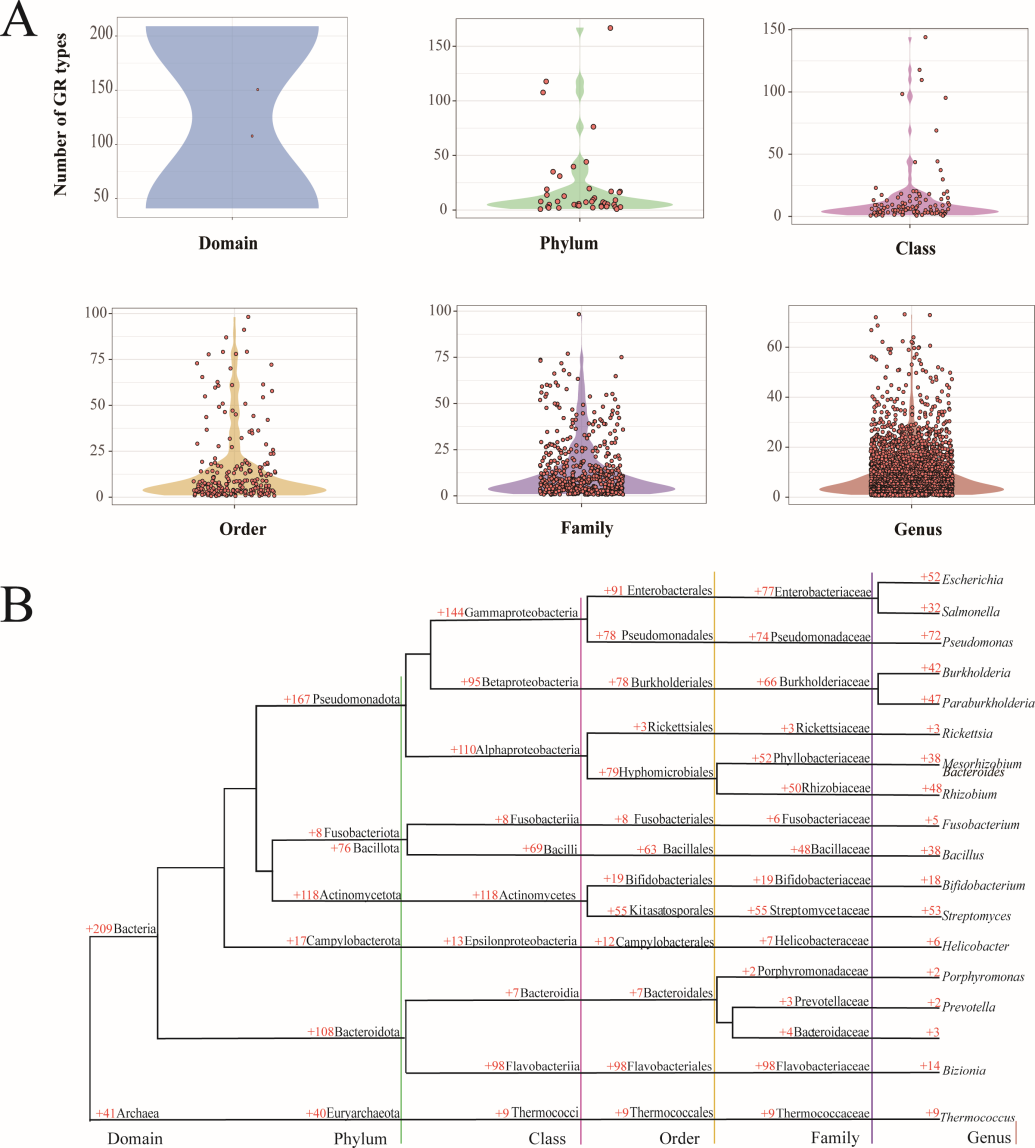
Phylogenetic distribution of the number of GR types across 14,800 bacterial and archaeal type strain genomes. (**A**) Violin plot representation of the number of GR types across bacterial and archaeal type strain genomes. Data distribution is shown by their corresponding phylum, class, order, and genus, with medians and interquartile ranges highlighted. The width indicates data density, and the colors identify different groups. (**B**) Phylogenetic distribution of the number of GR types among 18 genera and their corresponding phylum, class, and order. Bacterial and archaeal taxa are displayed according to their phylogeny (left). The numbers on the tree branches represent the total number of GR types within different evolutionary lineages.

To investigate the taxonomic distribution of GRs, we quantified the number of GR types across 14,800 bacterial and archaeal type strain genomes, spanning 44 phyla, 110 classes, 231 orders, 551 families, and 2,801 genera. GR type counts varied substantially across taxonomic ranks, ranging from 1 to 167 at the phylum level, from 1 to 144 at the class level, from 1 to 98 at both the order and family levels, and from 1 to 73 at the genus level (Fig. 3A). Despite this broad numerical range, density analysis revealed a strong concentration of taxa within lower GR-type intervals. Most genera encoded fewer than 25 GR types, while the majority of orders and families fell within the range of 10–30. These observations highlight the uneven and lineage-specific nature of regulatory complexity across prokaryotes.

To further characterize GR distribution in an evolutionary context, we performed 16S rRNA-based phylogenetic mapping of 18 representative genera (Fig. 3B). GR abundance exhibited marked variation across the phylogenetic tree. At the phylum level, Pseudomonadota harbored the largest number of GR types (167), followed by Actinomycetota (118), Bacteroidota (108), and Bacillota (76). In contrast, Fusobacteriota and Campylobacterota encoded only 8 and 17 GR types, respectively. At the genus level, facultative pathogens such as *Pseudomonas* (72), *Escherichia* (52), and *Salmonella* (32) exhibited high GR diversity. Environmental and symbiotic genera, including *Paraburkholderia* (47), *Burkholderia* (42), *Rhizobium* (48), and *Mesorhizobium* (38), also maintained relatively rich GR repertoires. *Streptomyces* encoded 53 GR types, consistent with its complex regulatory architecture linked to secondary metabolism. In contrast, genera such as *Rickettsia*, *Fusobacterium*, and *Thermococcus* encoded nine or fewer GR types, reflecting streamlined genomes and niche-specific adaptations. These results underscore phylogenetically structured patterns in GR distribution shaped by ecological and evolutionary pressures.

### Lineage-specific enrichment of GRs

**Fig 4:**
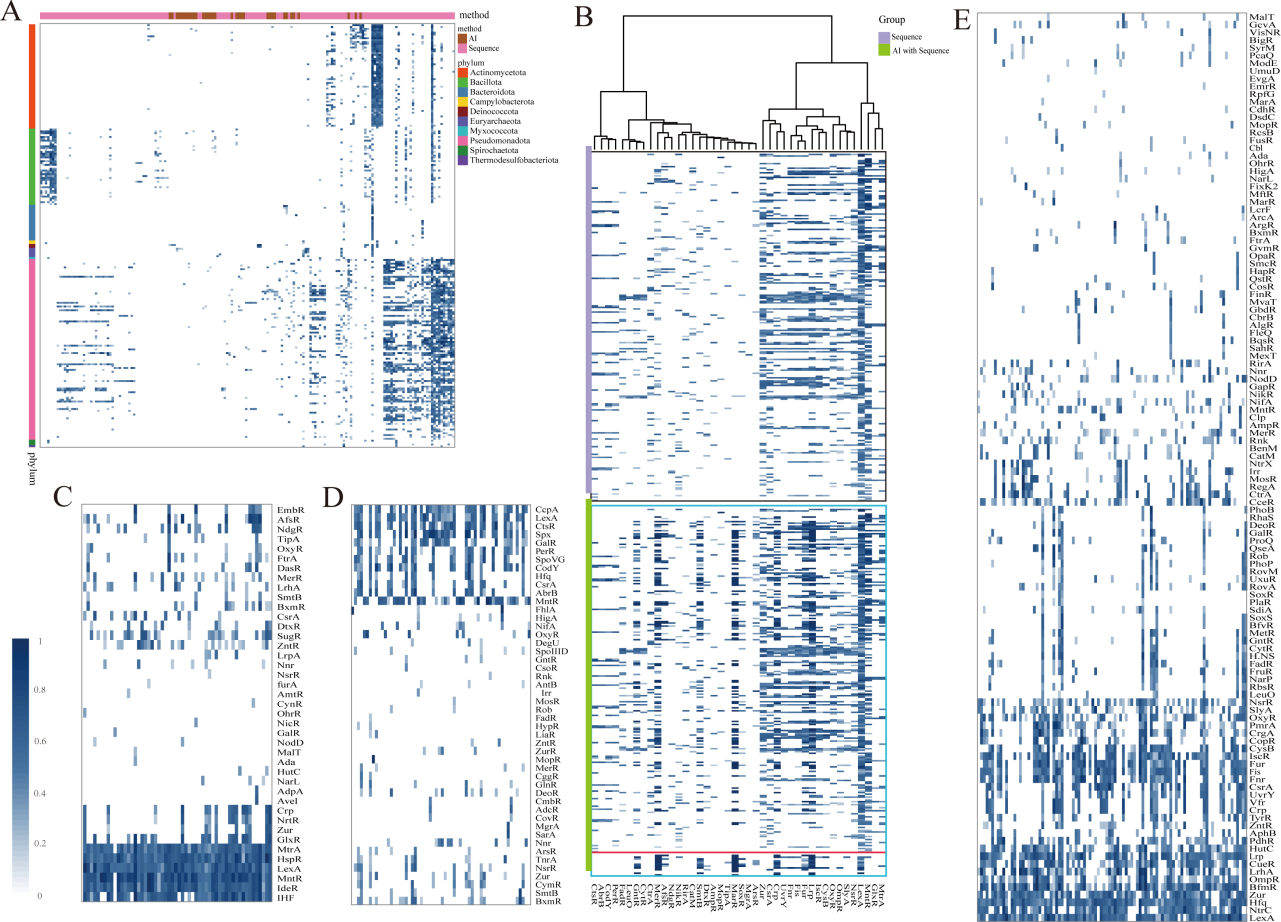
Lineage-specific enrichment of GRs. (**A**) Distribution of 214 experimentally validated GRs together with 52 putative novel GRs predicted by AI across major bacterial phyla. The lineage-specific enrichment patterns of conserved regulators are largely maintained. (**B**) Distribution of 173,256 homologs identified by AI compared with their corresponding validated GRs. The inclusion of homologs markedly increases overall abundance within major GR families but does not alter phylogenetic distribution profiles. Regulators such as GntR, MerR, SmtB, MarR, Crp, Lrp, and LexA become more prominent while retaining lineage-specific signatures. (**C–E**) Heatmap showing the abundance of GRs enriched in *Actinomycetota* (**C**), *Bacillota* (**D**), and *Pseudomonadota* (**E**), highlighting lineage-specific regulatory patterns and conserved functional modules.

To investigate lineage-specific enrichment, we analyzed GR-type distributions across major bacterial phyla (Fig. 4). Figure 4A illustrates the distribution of 214 experimentally validated GRs, together with 52 putative novel GRs predicted by AI, which largely mirror the lineage-specific patterns of conserved regulators. To further test the robustness of these patterns, we incorporated 173,256 homologs identified by AI alongside the validated GRs (Fig. 4B). While the inclusion of these homologs markedly increased overall abundance within major GR families, the phylogenetic distribution profiles remained stable. This amplification effect was particularly evident for regulators such as *GntR*, *MerR*, *SmtB*, *MarR*, *Crp*, *Lrp*, and *LexA*, which became more prominent without altering lineage-specific signatures.

Clustering analyses across phyla further revealed distinct sets of conserved GRs enriched in specific lineages, consistent with vertical inheritance and phylum-level regulatory specialization. In Actinomycetota, regulators such as *AfsR*, *SugR*, *NdgR*, *IdeR*, *MntR*, *LexA*, *HspR*, *MtrA*, and *IHF* dominated, reflecting control over secondary metabolism, development, and metal homeostasis (Fig. 4C). Bacillota showed enrichment of *CodY*, *CcpA*, *AbrB*, *PerR*, *SpoVG*, *CtsR*, and *SpxA*, which are central to nutrient sensing and sporulation (Fig. 4D). In Pseudomonadota, a phylogenetically diverse group encompassing both environmental and pathogenic taxa, regulators including *BfmR*, *Hfq*, *LrhA*, *OmpR*, *HutC*, *UvrY*, *CrgA*, and *SlyA* were associated with virulence, quorum sensing, and metabolic versatility (Fig. 4E). Collectively, these results demonstrate that AI-predicted homologs reinforce lineage-specific enrichment patterns and highlight the evolutionary stability of global regulatory repertoires across prokaryotic phyla.

### Diverse regulatory functions of GRs

We observed extensive functional divergence in GR regulons across genomes. Genome-wide binding site prediction revealed wide variation in the proportion and function of GR-regulated genes among lineages (Table S3). Core targets were defined as genes regulated in ≥50% of genomes from at least two genera. Core regulons showed minimal overlap across phyla, reflecting substantial regulatory divergence (Table S4).

**Fig 5:**
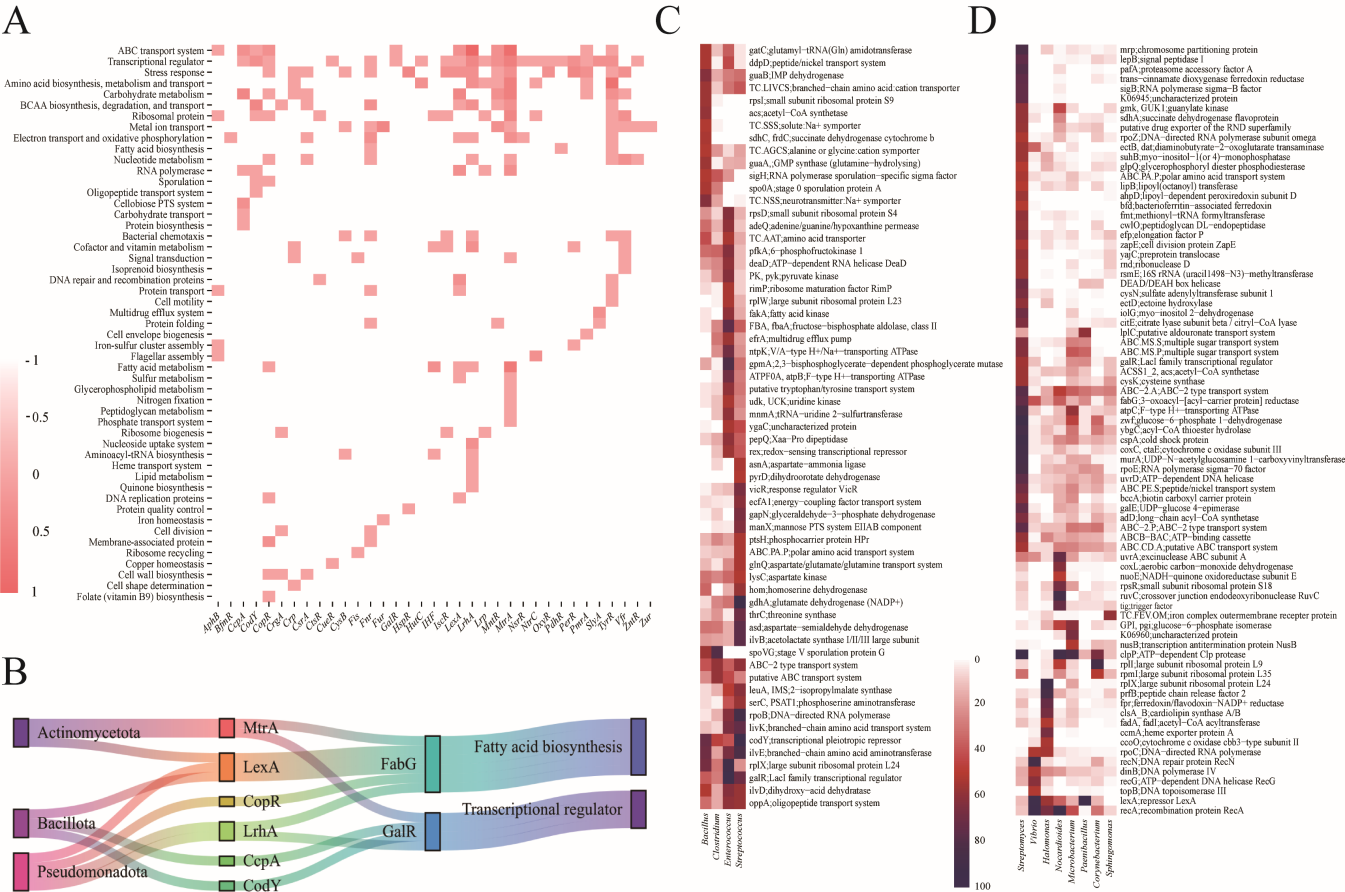
Functional diversity and convergence in GR target genes. (**A**) Heatmap of log_10_-transformed enrichment values showing core functional categories regulated by 35 phylum-level conserved GRs. (**B**) Sankey diagram illustrates convergence of multiple GRs on shared targets (*fabG* and *galR*) across *Actinomycetota*, *Bacillota*, and *Pseudomonadota*. (C and D) Genus-specific variation in *CodY* (**C**) and *LexA* (**D**) regulons, showing functional divergence in target gene composition despite conserved regulatory roles.

Analysis of 36 widely distributed GRs revealed a conserved regulatory core encompassing functions like ABC transport, amino acid and carbohydrate metabolism, ribosome biogenesis, metal ion transport, oxidative phosphorylation, and nucleotide metabolism (Fig. 5A; Table S5). These functions represent a conserved core regulatory module maintained across diverse lineages. Furthermore, cross-GR comparisons revealed instances of regulatory convergence, where different GRs regulate the same functional genes across or within phyla (Fig. 5B). For example, FabG, encoding a fatty acid biosynthesis enzyme, was found to be regulated by multiple GRs including *MtrA*, *LexA*, *CopR*, *LrhA*, *CcpA*, *CodY*, and *GalR* in *Actinomycetota*, *Bacillota*, and *Pseudomonadota*. Similarly, *GalR*, a regulator of carbohydrate metabolism, was itself targeted by several other GRs, suggesting the existence of shared regulatory hierarchies.

Conversely, GR-specific targets included genes related to sporulation, vitamin metabolism, multidrug resistance, and flagellar assembly (Fig. 5A). These observations point to substantial differences in GR regulon composition across regulators and lineages. To provide quantitative support, we further analyzed the proportion of functional target genes at the genus level for each regulator (Table S6). This analysis highlights clear differences in the genomic proportion of GR-regulated functions across lineages, underscoring the functional divergence of GRs in shaping lineage-specific regulatory programs. Comparative analysis of *CodY* and *LexA* regulons across genera illustrated this divergence (Fig. 5C and D). *CodY* maintained a conserved core related to nitrogen metabolism but regulated distinct sets of genes among genera, particularly those associated with branched-chain amino acid (BCAA) biosynthesis and peptide transport (Fig. 5C). *LexA* consistently targeted *recA* and *lexA* across taxa, but its regulation of other DNA repair genes varied substantially by genus (Fig. 5D)

### GRs operate in hierarchical and lineage-specific networks

**Fig 6:**
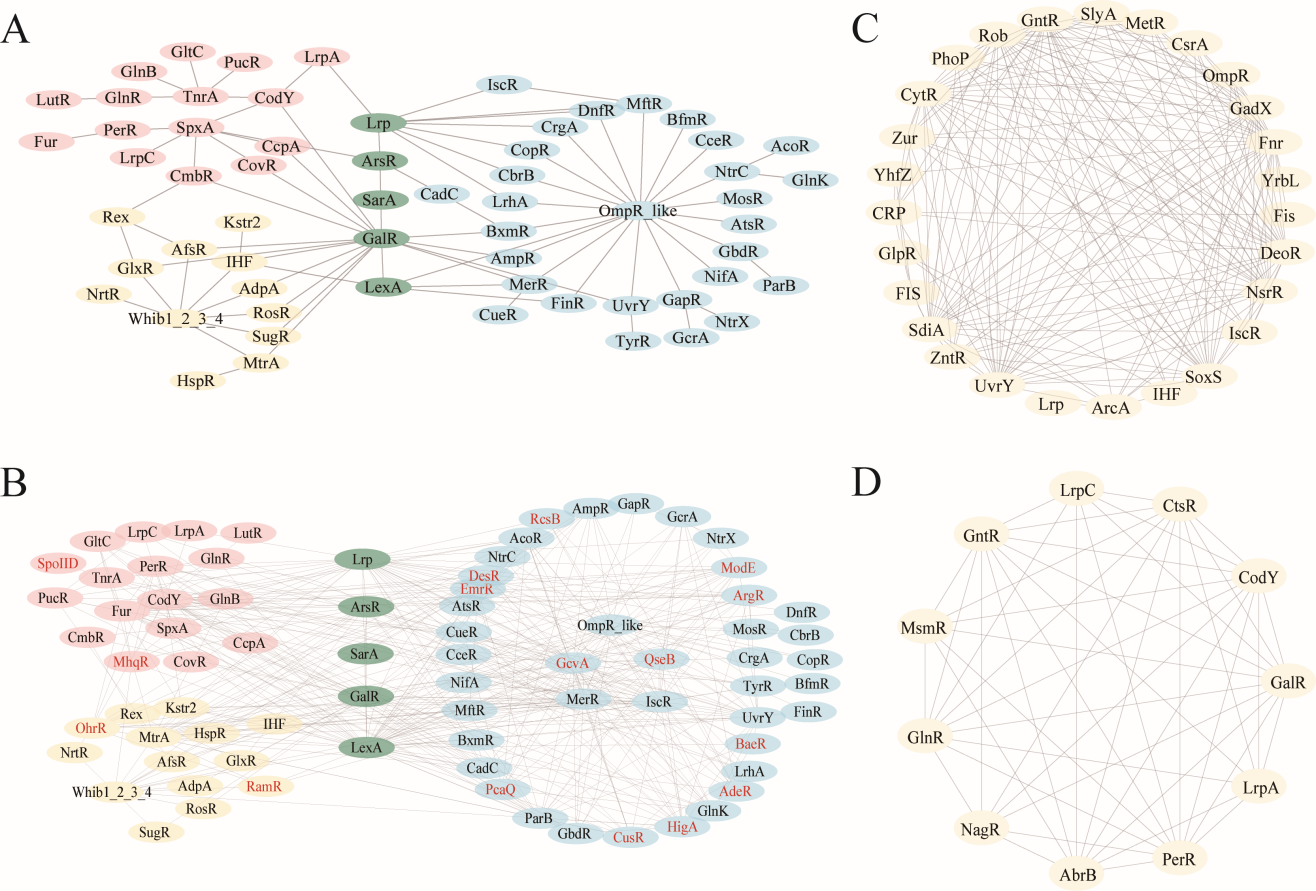
Network analysis of global regulators (GRs). (**A**) Baseline GR–GR interaction networks constructed from 214 validated GRs and their homologs across *Bacillota*, *Pseudomonadota*, and *Actinomycetota*, showing both shared central regulators (e.g., GalR) and lineage-specific hubs. (**B**) Expanded networks incorporating AI-predicted additional homologs of the 214 validated GRs together with 52 putative GRs, revealing new associations and novel hubs (e.g., QseB) and underscoring the increased connectivity and complexity of GR regulatory hierarchies. (**C** and **D**) Species-level GR networks for *Escherichia coli* (**C**) and *Bacillus subtilis* (**D**), illustrating dense cross-regulatory interactions. Nodes represent GRs; edges indicate predicted regulatory relationships. The networks reflect both conserved and lineage-specific architecture.

Network analysis of GRs in *Bacillota*, *Pseudomonadota*, and *Actinomycetota* was constructed from 214 well-characterized GRs and their predicted homologous proteins (Fig. 6A). Regulatory edges were defined when a GR was predicted to target another GR, thereby establishing direct GR–GR interactions. These baseline networks revealed that GRs operate within complex, highly interconnected systems rather than as isolated elements. Shared GRs such as *GalR* occupy central positions across all three phyla, reflecting their conserved role in coordinating core cellular functions. In contrast, lineage-specific GRs form distinct regulatory hubs that modulate phylum-enriched pathways. For instance, in *Bacillota*, *CodY*, *TnrA*, and *SpxA* exhibit strong connectivity and are linked to nitrogen metabolism, stress response, and sporulation. In *Pseudomonadota*, *OmpR*, *HigA*, and *ModE* are associated with environmental sensing and virulence-related regulation. In *Actinomycetota*, regulators such as *AfsR*, *WhiB*-family members, and *OhrR* are involved in stress adaptation and secondary metabolism.

To further extend this framework, we reconstructed an expanded network that incorporated additional homologs identified in 214 GRs together with the 52 putative GRs predicted by our AI-based approach (Fig. 6B). Compared with the baseline networks, the expanded network revealed new regulatory associations among canonical GRs and integrated novel GR nodes into the system. Notably, several putative GRs, including QseB and GcvA, emerged as central hubs, indicating that AI-identified regulators can occupy pivotal positions within global regulatory hierarchies. The expanded network also displayed markedly greater complexity and connectivity, underscoring how the inclusion of novel GRs reshapes the architecture of prokaryotic transcriptional regulation.

Furthermore, species-level networks constructed for *Escherichia coli* and *Bacillus subtilis* (Fig. 6C and D) further illustrate the dense cross-regulatory architecture among GRs. In *E. coli*, major regulators such as *CRP*, *ArcA*, *Fnr*, *Fur*, and *H-NS* form an integrated core governing metabolic and stress responses. In *B. subtilis*, GRs including *CodY*, *CcpA*, *AbrB*, and *PerR* define a similarly structured network controlling nutrient sensing and sporulation. Collectively, these results indicate that GRs act as hubs embedded in hierarchical and modular regulatory networks, supporting global signal integration and enabling context-dependent regulatory specificity across taxa.

### PGRR database

**Fig 7:**
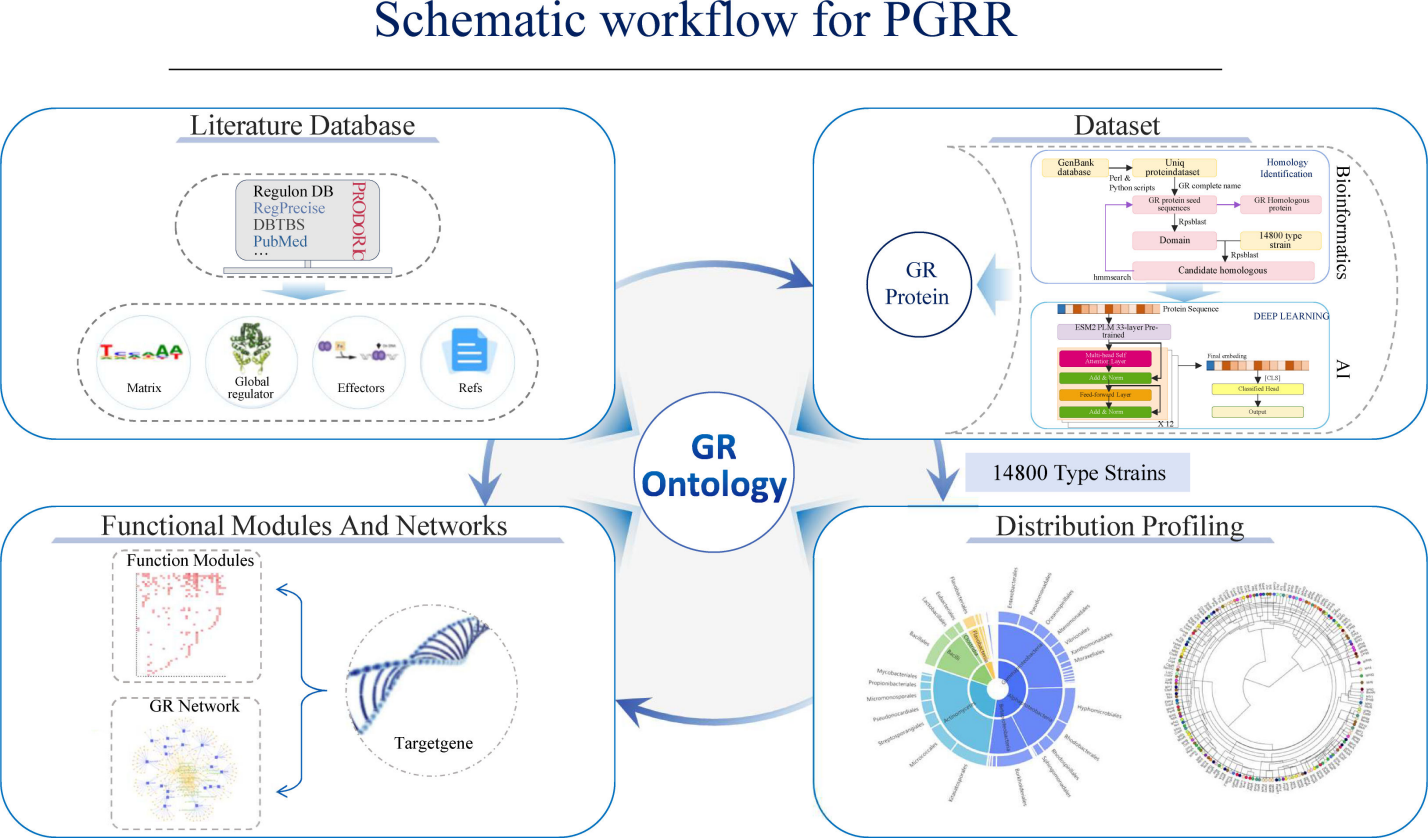
Overview of the PGRR database construction pipeline. Data were collected from literature and public databases and analyzed using RPS-BLAST, HMMER, and the ESM2 model. GRs were identified across 14,800 type strain genomes, and their predicted targets, regulatory networks, and functional annotations were integrated into the PGRR platform.

The PGRR (https://nmdc.cn/pgrr/) is an integrated platform that consolidates both experimentally validated and AI-predicted GRs. It provides comprehensive data on the taxonomic distribution, functional roles, and regulatory networks of GRs across 14,800 type strain genomes (Fig. 7). By combining state-of-the-art bioinformatics pipelines with deep learning models, PGRR enables systematic exploration of GR-mediated regulatory architectures in prokaryotes. Beyond its utility for microbial systems biology, PGRR serves as a foundational resource for synthetic biology. It facilitates the identification and functional repurposing of GRs as genetic control elements, supporting the rational design of synthetic regulatory circuits and the engineering of microbial chassis with customized transcriptional programs.

## DISCUSSION

This study presents a comprehensive and scalable framework for the discovery and characterization of GRs across prokaryotic genomes, offering new insights into their evolutionary conservation, functional divergence, and regulatory complexity. By integrating pLMs with deep learning architectures, we expanded the known GR repertoire, identifying over 270,000 previously unrecognized homologs, including 52 putative GRs. This scale of discovery goes beyond the reach of traditional sequence similarity or domain-centric methods (26–28), highlighting the advantages of AI-driven approaches for detecting remote homologs and uncovering cryptic regulatory elements (32, 34, 35). A notable strength of our model is its capacity to generalize beyond known GRs. Among these 52 putative GRs, 39 GRs are supported by literature or predicted target genes, reinforcing the robustness and biological relevance of the deep learning framework (35, 36). AI-predicted GRs such as GcvA, OhrR, DesR, EmrR, and HigA were consistently found to be central nodes within regulatory networks, further validating their functional importance. These findings not only broaden the scope of known GRs but also establish a generalizable framework for discovering other classes of regulatory proteins, including transcription factors, sigma factors, and non-coding RNAs (36, 38).

Our analyses highlight that GRs function not as isolated entities but as integral components of highly interconnected regulatory networks. These networks are hierarchical and modular, coupling deeply conserved functions with lineage-specific innovations. Canonical regulators such as *GalR* consistently occupy central positions, underscoring their essential role in maintaining core physiological control, whereas lineage-enriched hubs illustrate the evolutionary flexibility of regulatory repertoires. Importantly, by incorporating homologs and AI-predicted GRs, we revealed an expanded and more intricate architecture in which previously uncharacterized regulators—including *GcvA* and *QseB*—emerged as network hubs. This underscores that novel GRs may exert global influence comparable to established regulators, thereby broadening the functional landscape of transcriptional control and providing prioritized candidates for future validation. At the species level, the dense cross-regulatory architectures exemplified by *E. coli* and *B. subtilis* demonstrate how GRs integrate environmental and metabolic signals into modular circuits, operating as hubs rather than independent switches. Collectively, these findings emphasize that GR function is best understood in a network context and demonstrate the power of AI-driven comparative genomics and predictive modeling to uncover hidden layers of prokaryotic regulation. Our functional analysis of GR targets highlights that global regulation is a complex and integrated process. Different GRs may converge on common targets, while the same GR can control divergent modules across lineages, reflecting both cooperation and lineage-specific rewiring. A notable example is the fabG gene, encoding 3-ketoacyl-acyl carrier protein reductase (39). We found that fabG is regulated by multiple GRs involved in stress adaptation, metabolism, and redox balance, underscoring how a single metabolic hub integrates diverse signals. This convergence, conserved across taxa, emphasizes the functional diversity and interconnectivity of GRs, consistent with the critical role of membrane lipid composition in bacterial survival and stress resilience (40). Beyond fabG, regulators such as LexA and CodY illustrate how ecological pressures drive lineage-specific rewiring of conserved regulons (4, 41, 42). Such plasticity reflects the inherent complexity of prokaryotic regulatory systems. From a synthetic biology perspective, the GRs identified here represent a versatile toolkit, with extremophilic or understudied taxa offering novel mechanisms for regulation under stress or conditions (13–15).

To facilitate community-wide use, we established PGRR, an open-access database that integrates GR sequences, phylogenetic distributions, predicted targets, and interaction networks, thereby providing the most comprehensive catalog of global regulators currently available for comparative and applied research. Beyond this resource, the methodology presented here offers a generalizable framework for mapping broader classes of regulatory proteins across prokaryotes. Future integration of deep learning-based predictions with high-resolution experimental approaches such as ChIP-exo and DAP-seq (20, 21) will further refine network-level annotations and expand functional insights. As an evolving platform, PGRR is positioned to support advances in microbial systems biology and genome-scale regulatory modeling while also serving as a foundation for the rational design of next-generation synthetic biology tools (36, 43–45).

Our deep learning framework was deliberately designed to address the closed-set nature of classification by distinguishing GRs from non-GRs, rather than assigning sequences to a fixed set of predefined GR types. In doing so, the model learns common sequence and structural features shared across diverse GR families, which allows it to capture generalizable properties of GRs. The specific GR type of a predicted sequence is subsequently annotated through comparative bioinformatics analyses and integration with curated resources. This design enables the discovery of GR-like proteins that extend beyond existing classifications; sequences that do not match any known GR type in PGRR are designated as putative novel GRs, thereby providing candidates for further investigation. Nevertheless, the framework also has limitations. The ability to recognize novel GRs is constrained by the diversity of training data, and highly divergent GRs that do not share sufficient common features with known families may remain undetected. In addition, prediction bias and false positives are inherent challenges in any classification system. Importantly, the flexibility of our approach allows for continuous refinement: as more GRs are experimentally validated, the model can be iteratively retrained, thereby progressively improving its accuracy, generalizability, and coverage across prokaryotic diversity.

### Conclusion

This study expands the repertoire of prokaryotic GRs by integrating machine learning with large-scale comparative genomics. The resulting PGRR database offers the most comprehensive resource of GR sequences, taxonomic distributions, predicted functions, and regulatory networks to date. Together, these resources advance our understanding of microbial regulation and provide a versatile toolkit for systems biology, regulatory modeling, and synthetic biology. In addition, the framework developed here serves as a practical tool—GR**-**Discriminator—for the discovery of prokaryotic GRs (https://nmdc.cn/pgrr/tools/discriminator).

## MATERIALS AND METHODS

### Data collection and integration of GRs

A dual-strategy framework was employed to curate a comprehensive list of GRs for inclusion in the PGRR. First, a systematic search was conducted across established regulatory databases, including PRODORIC (https://www.prodoric.de), the DBD database (46), and RegPrecise (http://regprecise.lbl.gov), to retrieve experimentally validated or computationally predicted GRs. To complement this, a literature-based curation was performed using PubMed, applying targeted search terms such as “bacterial regulatory factor,” “archaeal regulatory factor,” “DNaseI footprint,” “electrophoretic mobility shift assay,” and “ChIP-seq & prokaryotes.”. The combined database and literature mining strategy yielded 214 non-redundant GRs.

To further annotate these GRs, binding site motifs and cofactor associations were manually curated from peer-reviewed literature and four specialized motif databases: CollecTF (http://collectf.umbc.edu), PRODORIC (https://www.prodoric.de), RegTransBase (47), and GroovDB (https://groov.bio). Additional features, including GR family classification, regulatory mechanism (activator, repressor, dual), and associated biological functions, were extracted from the same sources. Seed protein sequences for each GR were retrieved from the National Center for Biotechnology Information RefSeq database (https://www.ncbi.nlm.nih.gov/refseq/).

### Identification of homologous proteins for 214 experimentally validated GRs based on bioinformatics

To identify homologous proteins corresponding to 214 experimentally validated GRs across 14,800 prokaryotic type strain genomes, a two-step homology prediction pipeline was applied. Initially, seed protein sequences were retrieved from GenBank, and protein domains were annotated using the Conserved Domains Database (https://www.ncbi.nlm.nih.gov/Structure/cdd/cdd.shtml). For GRs exhibiting unique domain signatures, a Reverse Position Specific BLAST search was performed against a comprehensive protein data set derived from the genomes of the 14,800 type strains. Candidate proteins sharing conserved GR domains were retained. Subsequently, HMMER (v.3.1b2) was used to perform an hmmsearch with supervised HMMs built from GR seed sequences to detect distant homologs with high sensitivity.

### Identification of homologous proteins of all GRs based on AI model data set preparation

A comprehensive data set was constructed comprising 91,604 experimentally validated or curated GR protein sequences spanning 169 distinct GR types, along with 21,518,895 non-GR sequences classified into 16 functional categories. To evaluate the model’s generalization ability on unseen GR types, 27 GR types were reserved and excluded from model training. These types, selected based on sequence abundance (excluding types with <10 sequences), were evenly partitioned into validation and test sets (50% each). For the remaining 142 GR types, 20% of sequences from each type were randomly assigned to validation and test sets (10% each), with the remaining 80% used for model training. Non-GR sequences were stratified and randomly assigned to training, validation, and test sets using an 8:1:1 ratio to ensure balanced representation across functional categories. TFs, which are closely related to GRs but do not meet strict GR criteria, were labeled separately as a distinct non-GR category to serve as hard negative controls.

### Model architecture

We utilized the ESM2 protein language model (650M parameters; model: esm2_t33_650M_UR50D, embedding dimension: 1280) as the feature extraction backbone. A custom GR classifier module was constructed on top of ESM2, consisting of a 12-layer transformer encoder with 512-dimensional hidden layers. Each layer incorporated an eight-head multihead attention mechanism (channel dimension: 64) and a position-wise feed-forward network (inner dimension: 512), with residual connections followed by layer normalization applied throughout.

### Model training

During training, the ESM2 backbone was frozen to prevent overfitting and reduce computational complexity. The GR classifier was optimized using a learning rate of 1 × 10⁻⁶ and a warm-up strategy over the first 1,000 batches. Additional training strategies included gradient clipping (maximum norm: 100), dropout (rate: 0.4), and weight decay (1 × 10⁻¹) to promote model generalization. To address class imbalance, negative samples were randomly subsampled at each epoch and combined with all available positive GR sequences, ensuring exposure to a diverse array of negative examples. To further emphasize difficult-to-distinguish cases, TF-labeled non-GRs were assigned a fivefold higher sample weight during training. This strategy enabled sensitive and specific detection of homologous GR proteins beyond conventional alignment-based methods.

### Binding site scanning and target gene prediction

To identify binding sites of GRs, a total of 186 experimentally validated or literature-supported binding motifs corresponding to 172 GRs were compiled from multiple databases and primary literature sources. For each homologous GR identified within a genome, a 400-nucleotide sequence upstream of the transcription start site of all genes was extracted. Binding site scanning was performed using FIMO (v.5.5.2), which searched for occurrences of GR-specific binding motifs within these upstream sequences. To minimize false positives inherent to PWM-based motif scanning, we employed a three-tiered filtering strategy. First, all sequences were screened using FIMO with its default *P* value threshold of 1 × 10⁻⁴. Second, for extremely short motifs (<6 bp), the *P* value threshold was stringently set to zero to further suppress spurious matches. Third, candidate motifs were manually inspected to evaluate sequence symmetry and complementarity, ensuring that only biologically plausible motifs were retained. Genes located immediately downstream of the predicted binding motifs were designated as candidate GR target genes.

### Functional annotation and regulatory network construction

Annotation of predicted GR target genes was performed by querying the Non-Redundant Protein Sequence Database, Comprehensive Antibiotic Resistance Database, Virulence Factor Database, Kyoto Encyclopedia of Genes and Genomes, and Clusters of Orthologous Genes. Genes were grouped into functional modules based on these annotations. Core target genes were defined using a hierarchical approach: (i) at the genus level, genes present in ≥50% of genomes within a genus were designated as genus-level core targets; (ii) genes that satisfied the genus-level core criteria across two or more genera were further classified as core GR targets; and (iii) if no targets met these criteria, the top five genes with the highest prevalence across genomes were selected as core targets. Regulatory interactions between GRs were inferred by identifying cases where one GR was predicted to regulate another. These interactions were used to construct phylum-level and species-level GR interaction networks. Network visualization and topological analysis were conducted using Cytoscape (v.3.10.2), allowing exploration of GR interaction patterns and functional roles across taxonomic groups.

### The PGRR database

The PGRR is an integrated platform that consolidates comprehensive information on GRs across prokaryotic genomes. GR data were collected from curated databases such as RegulonDB (https://regulondb.ccg.unam.mx), RegPrecise (http://regprecise.lbl.gov), and DBTBS (http://dbtbs.hgc.jp), as well as through systematic literature mining. The database is structured into four major components: (i) the Literature-Derived GR Repository module contains manually curated annotations for each GR, including regulatory family classification, DNA-binding motifs, and predicted or experimentally validated target genes; (ii) the GR Protein Sequence Collection provides access to a non-redundant set of GR protein sequences, serving as reference seed sequences for homology-based prediction and classification; (iii) GR Distribution Profiling maps the occurrence of GRs across 14,800 representative type strain genomes, enabling comparative analysis of taxonomic distribution, conservation patterns, and ecological associations; and (iv) Functional Modules and Regulatory Network Architecture integrates GR-target gene relationships to reconstruct regulatory and metabolic networks. This component supports visualization of regulatory hierarchies and interaction pathways, facilitating insights into the functional roles of GRs in cellular processes and microbial adaptation. PGRR is publicly available at https://nmdc.cn/pgrr/, providing a centralized resource for GR discovery, comparative genomics, and synthetic biology applications.

## SUPPLEMENTAL MATERIAL

Supplemental Tables msystems.00950-25-s0001.xlsx Tables S1–S6.

ASM does not own the copyrights to Supplemental Material that may be linked to, or accessed through, an article. The authors have granted ASM a non-exclusive, world-wide license to publish the Supplemental Material files. Please contact the corresponding author directly for reuse.
